# Suture Closure versus Non-Closure of Subcutaneous Fat and Cosmetic Outcome after Cesarean Section: A Randomized Controlled Trial

**DOI:** 10.1371/journal.pone.0114730

**Published:** 2014-12-10

**Authors:** Heinrich Husslein, Martina Gutschi, Heinz Leipold, Christoph Herbst, Maximilian Franz, Christof Worda

**Affiliations:** 1 Department of Obstetrics and Gynecology, Klinikum Klagenfurt am Woerthersee, Feschnigstrasse 11, 9020 Klagenfurt, Austria; 2 Department of Obstetrics and Gynecology, Medical University Vienna, Waehringer Guertel 18–20, 1090 Wien, Austria; Oslo University Hospital, Ullevål, Norway

## Abstract

**Introduction:**

To investigate the effect of subcutaneous fat suture closure versus non-closure at cesarean section (CS) on long-term cosmetic outcome.

**Material and Methods:**

Women undergoing planned or unplanned CS were randomized to either subcutaneous fat suture closure or non-closure using a 1∶1 allocation algorithm. Participants and outcome assessors were blinded to group allocation. Scar evaluation was performed after two and six months. Primary outcome measures were Patient and Observer Scar Assessment Scale (POSAS) summary scores six months after surgery. Secondary outcome measures were Vancouver Scar Scale (VSS) summary scores, retraction of the scar below the level of the surrounding skin, duration of surgery, and development of hematoma, seroma, surgical site infection (SSI) or wound disruption. Data were analyzed according to the intention to treat principle.

**Results:**

A total of 116 women were randomized and 91 participants, 47 in the closure and 44 in the non-closure group, completed the trial and were analyzed. There were no differences in patient morphometrics or surgery indications between groups. At two and six months no significant differences were found with respect to POSAS or VSS scores between groups. After two months significantly more women in the non-closure group described their scar as being retracted below the level of the skin (36% vs. 15%, p = 0.02) whereas no difference was observed at six months. There were significantly more hematomas in the non-closure (25%) compared to the closure group (4%) (p = 0.005). There was no difference in duration of surgery, SSI, seroma formation or wound disruption between groups.

**Conclusions:**

Suture closure of the subcutaneous fat at CS does not affect long-term cosmetic outcome. (Level I evidence).

**Trial Registration:**

ClinicalTrials.gov NCT01542346.

## Introduction

Each year cesarean sections (CS) are performed in millions of women worldwide, typically resulting in significant skin scaring. Cosmetic outcome of the scar is important, since it is the only visible stigmatization after CS. It is often overlooked that scars can cause considerable psychosocial distress, which seems to be most closely related to patient-rated scar severity and scar location [1]. Due to the high frequency of CS and these potential long-term consequences, it is essential to determine the surgical technique rendering optimal cosmetic results.

As with most surgical procedures, there is no standard technique for CS [2]–[3]. A variety of techniques exist for nearly every step of the procedure, many of which have been studied [4]–[6]. The result is a large variation in the choice of surgical method chosen by the operator. Different endpoints have been studied when comparing different wound closure techniques, including wound complication rates, short-term cosmetic outcome and long-term cosmetic outcome [3], [7]. To date there is insufficient evidence to favor one method of skin closure [3]. In most studies investigating cosmetic outcome non-absorbable staples have been compared to different types of absorbable subcuticular sutures and cosmetic outcome was similar [3]. Several studies have investigated the effect of closure of the subcutaneous fat on wound complication rates, however there is insufficient data regarding the influence of subcutaneous suture closure on wound cosmesis [7], [8]. The available studies report conflicting results, short-term cosmetic outcomes only or have methodological flaws [9]–[12].

The cosmetic outcome after closure of the subcutaneous fat is nevertheless worth being studied. Although the development of hypertrophic scarring seems to have multiple causes, tension acting on a scar has been reported to be a common initiating factor [13], [14]. Next to closure of the dead space, closure of the subcutaneous fat theoretically decreases tension on the above skin layer. Suture closure of the subcutaneous fat could therefore result in superior cosmetic outcome by decreasing tension on the skin layer. Further, suture closure of the subcutaneous fat theoretically reduces the rate of postoperative hematoma and seroma development and might prevent retraction of the scar below the level of the surrounding skin, by closing off dead space and achieving optimal leveling of the opposing wound sides. On the other hand suture closure of subcutaneous tissue leads to an inflammatory reaction against the suture material and could therefore result in poorer cosmesis.

Although evidence suggests that suture closure of the subcutaneous fat at the time of CS reduces the risk of wound disruption in women with a subcutaneous tissue larger than two centimeters [8], the effect of this intervention on cosmetic outcome is not sufficiently studied. This might lead to reluctance to routinely implement this important intervention. Therefore, the aim of this study was to investigate the effect of interrupted suture closure of the subcutaneous fat on long-term cosmetic outcome after CS.

## Materials and Methods

This randomized, controlled trial was performed at the Klinikum Klagenfurt am Woerthersee, Austria, in collaboration with the Medical University Vienna, Austria. The study was approved by the local institutional review board, Ethikkommission des Landes Kaernten (A16/10), and was registered with the clinical trials registry (Clinical-Trials.gov Identifier NCT 01542346). The protocol for this trial, supporting CONSORT checklist and the data underlying the results of this trial are available as supporting information; see S1 Checklist, S1 Protocol and S1 Data.

From March 2012 until July 2013 women who were planning a delivery at our institution were invited to participate in this trial. Pregnant women between 18 and 45 years, of Caucasian origin and literate in German language were eligible for study inclusion. Exclusion criteria included clinical signs of infection at the time of CS, HELLP syndrome or preeclampsia, history of keloids, previous transverse suprapubic scars and a medical disorder that could affect wound healing, such as known hypersensitivity to any of the suture materials used in the protocol, diabetes mellitus, disorders requiring chronic corticosteroid use or immunosuppression. Women planning to deliver at our institution are typically seen between 36 and 38 weeks of gestation. At this visit eligible women were invited to participate by one of the research team members. The trial was explained and written informed consent was obtained. We chose to recruit women between 36 and 38 weeks of gestation because we aimed to include all indications for CS. Recruitment, including explanation of the trial and informed consent at the time of CS indication would be impossible in cases of emergency CS and probably ethically inadequate in cases of failure to progress or non-reassuring fetal status.

Women undergoing CS were randomized by the attending midwife after skin incision, by drawing a sealed envelope containing the information regarding group allocation (ie, group A: suture closure of the subcutaneous fat or group B: non-closure of the subcutaneous fat). The envelope was shown to the surgeon and the operative nurse and not read out loud to maintain blinding of the participants. The envelopes were consecutively numbered according to the sequence of a computer-generated randomization plan using one-to-one randomization.

All cases received a single dose of intravenous cefuroxime (1500 mg) as perioperative single-shot antibiotic prophylaxis, when possible prior to skin incision. CS was performed by staff physicians or senior residents (n = 8), all of whom had performed at least 50 CS. The CS was performed according to the modified Misgav Ladach technique [15].

In women randomized to the closure group (group A), the subcutaneous fat was closed with three to five interrupted, Polysorb 3–0 sutures using a V 26 needle [16]. The sutures were tied until the tissue was adequately re-approximated, but not as hard as possible to avoid necrosis. In women randomized to the control group (group B), subcutaneous fat was not suture closed. Participants were blinded to their group allocation. In all participants the skin was closed using non-absorbable staples; thin adhesive strips were not used at skin closure. The wound was dressed with an abdominal pad and adhesive tape. The length of surgery from skin incision to skin closure was recorded for each CS. We used staples for efficiency and because subcuticular skin closure does not improve long-term cosmetic outcome [3], [6]. The wound dressing was removed on postoperative day one. Staples were removed on postoperative day five to prevent wound disruption. Early postoperative ambulation and graduated stockings were used as methods of thromboprophylaxis.

On the day of discharge from hospital, presence and location of any hematoma surrounding the wound was noted. Patients were admitted until postoperative day five, which is a result of the medical system and not of patient care.

Participants were seen in follow-up after two and six months. At two months initial healing results were evaluated and additional data regarding SSI within 30 days of CS and any other adverse wound events (ie, seroma development, wound disruption, need for reclosure) were recorded [17]. At six months the final scar appearance was evaluated. At both follow-up appointments the cosmetic outcome of the CS scar was assessed using the following validated scar assessment tools: objective scar rating was performed using the Vancouver Scar Scale (VSS) [18], [19] and the observer component of the Patient and Observer Scar Assessment Scale (POSAS) [20], [21]. Subjective scar rating was performed using the patient component of the POSAS. Further, we asked participants about the self-perceived presence or absence of any scar retraction below the level of the surrounding skin. Objective scar assessment was performed by one observer blinded to the patients group allocation. For this purpose two of the authors (HH and MG) were available as observers. More than one observer was necessary to maintain observer blinding in the case that one of these observers was involved in the participants CS. High inter-rater reliability for every item of the observer component of the POSAS (intraclass correlation coefficients ranged from 0.85 to 0.95) has been demonstrated previously [21].

The study’s prespecified primary outcome measures were patient and observer POSAS summary scores six month after CS. Prespecified secondary outcome measures included POSAS summary scores after two months, VSS summary scores after two and six months, retraction of the scar below the level of the surrounding skin after two and six months, duration of surgery and development of hematoma, seroma, SSI or wound disruption. Wound complications and their treatment were assessed by self-report and chart review with proper adjudication of outcomes. Postoperative SSI was defined according to the Centers for Disease Control and Prevention guideline [17]. Wound disruption was defined as any skin separation that was large enough to admit a sterile cotton-tipped swab and hematoma was defined as any visible or otherwise diagnosed (ie, ultrasound) collection of blood surrounding the wound [22].

All participants who missed a follow-up appointment were reminded via telephone and in case further participation was declined, any specific reason for dropout was enquired.

At the time of study conception the only study reporting POSAS scores for CS scars was published by Cromi et al [6]. This study reported mean POSAS summary scores of 23.6 with a mean standard deviation of 7.6. Assuming a clinically significant effect would be evident if there is a 20% ( = 5 points) difference in mean POSAS summary scores and accounting for a mean standard deviation of 8, the sample size was calculated to be 41 participants for each arm applying α = 0.05 and 80% power. To account for an attrition rate of up to 40% a total of 116 patients were enrolled.

Statistical analysis was performed with SPSS software (version 21.0; SPSS, Chicago, IL) according to the intention to treat principle. Continuous variables are summarized as median (range), and categorical data as percentages. Skewed distribution was tested via Kolmogrov Smirnov test. Chi2 test, Fishers exact test and Mann-Whitney-U test were used accordingly. Additionally we performed an exploratory univariate variance analysis for repeated measurements using the different scores at 2 month and 6 month as within-subjects factors and closure group as between-subject factor. Effects of different scores were quantified with Cliffs delta [23], partial eta squared [24] or relative risks (RR), as appropriate. P values of ≤.05 were considered significant.

## Results

**Figure 1:**
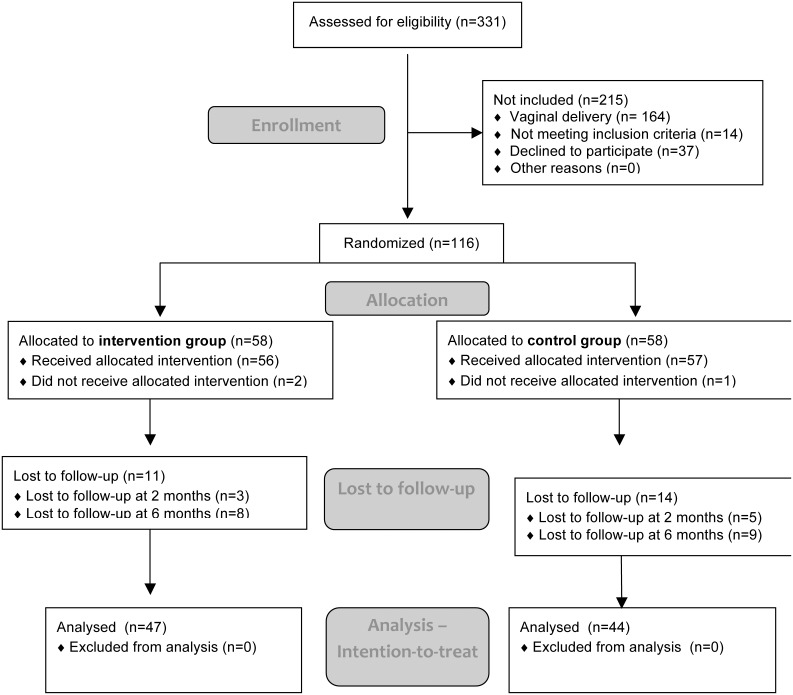
Flow diagram showing the progress through the trial.

Overall 331 women were assessed for eligibility, of whom 37 declined participation and 14 did not meet inclusion criteria. Of the 280 who were able and willing to participate in this trial, 116 underwent CS delivery and thus were randomized. All women were of Caucasian origin. A total of 25 participants (21,6%), 11 in the closure and 14 in the non-closure group were lost to follow-up (Fig. 1). There was no difference regarding any demographic or obstetric characteristics or intra- or postoperative complications in patients lost to follow-up compared to patients who completed the trial. A total of 91 participants, 47 in the closure and 44 in the non-closure group were available for final analysis. Three participants did not receive the correct intervention but were included in final analysis according to the intention to treat principle (Fig. 1). Baseline demographic and obstetric characteristics are shown in Table 1. There was no difference between the two groups regarding parity, maternal age, body-mass-index (BMI), smoking or indication for CS.

**Table 1:** Comparisons of baseline demographic and obstetrical characteristics between closure groups (n = 91).

	No subcutaneous sutureclosure (n = 44)	Subcutaneous sutureclosure (n = 47)
Maternal age (years)	30 (17–40)	28 (18–43)
Body-mass-index (kg/m^2^)	28.6 (17.4–39.2)	26.6 (19.6–39.7)
Smoking, n (%)	6 (14)	4 (8)
Indication, n (%)		
Cesarean section before labour	20 (45)	26 (55)
Cesarean section after labour	16 (36)	18 (38)
Emergency cesarean section	4 (9)	1 (2)
Cesarean section in multiple pregnancies	4 (9)	2 (4)

Values are given as median (range) or numbers (%).

Scar assessment at six month after CS revealed no significant difference between the two groups with respect to objective or subjective POSAS summary scores, VSS summary scores or patient self-rating on the presence or absence of a retraction of the scar below the level of the skin (Table 2).

**Table 2:** Results 6 month following cesarean delivery (n = 91).

	No subcutaneoussuture closure (n = 44)	Subcutaneoussuture closure (n = 47)	*Effect size*	*P*– value^†^
Scar retraction belowthe level of the skin	8 (18)	7 (15)	RR .82 (.32–2.07)	.67
VSS score	5 (0–11)	6 (1–11)	δ = .09	.47
POSAS score				
OSAS	19 (12–33)	19 (13–32)	δ = .06	.59
PSAS	17 (11–35)	17 (9–31)	δ = .04	.71

Values are given as median (range) or numbers (%), ^†^Mann Whitney U test or chi square test. *RR,* Relative Risk (95% Confidence Intervall); *δ*, Cliffs Delta; *OSAS*, Observer Scar Assessment Scale; *POSAS*, Patient and Observer Scar Assessment Scale; *PSAS*, Patient Scar Assessment Scale; *VSS*, Vancouver Scar Scale.

Scar assessment after two months revealed no difference in objective and subjective POSAS summary scores or VSS summary scores (Table 3). However, patient self-rating on the presence or absence of a retraction of the scar below the level of the skin was significantly less in the closure group compared to the non-closure group (Table 3).

**Table 3:** Results at discharge and 2 months following cesarean delivery (n = 91).

	No subcutaneoussuture closure (n = 44)	Subcutaneoussuture closure (n = 47)	*Effect size*	*P*– value^†^
Hematoma	11 (25)	2 (4)	RR .17 (.04–.73)	.005
Wound disruption	1 (1)	0 (0)	n/a	1
Surgical site infection	3 (7)	1 (2)	RR .31 (.03–2.89)	.35
Scar retraction belowthe level of the skin	16 (36)	7 (15)	RR .41 (.19–.90)	.02
VSS score	7 (2–11)	7 (3–12)	δ = .14	.25
POSAS score				
OSAS	22 (14–34)	20 (14–33)	δ = .15	.2
PSAS	20 (13–47)	18 (12–39)	δ = .08	.53

Values are given as median (range) or numbers (%), ^†^Mann Whitney U test, Fisheŕs exact test or chi square test. *RR,* Relative Risk (95% Confidence Intervall); *δ*, Cliffs Delta; *n/a*, not applicable; *OSAS*, Observer Scar Assessment Scale; *POSAS*, Patient and Observer Scar Assessment Scale; *PSAS*, Patient Scar Assessment Scale; *VSS*, Vancouver Scar Scale.

Exploratory univariate variance analysis for repeated measurements demonstrated a significant decrease of mean objective and subjective POSAS summary scores between the two months and the six months follow-up (OSAS and PSAS; p = .002, partial eta^2^.09 and p = .005, partial eta^2^.09) but no significant difference according to subcutaneous closure groups (OSAS and PSAS; p = 0.44, partial eta^2^.02 and p = 0.5, partial eta^2^.003, respectively).

At discharge from hospital a significant higher proportion of wound hematoma in the non-closure group compared to the non-closure group was noted, however there was no significant difference with respect to SSI or wound disruption rate (Table 3). One hematoma was diagnosed via ultrasound (closure group). A total of three superficial and one deep SSI occurred (non-closure group), of which none required surgical intervention. There was no case of wound seroma. The deep SSI went on to develop wound disruption (BMI: 30.3), which was managed conservatively. One woman required a second surgical exploration due to postoperative bleeding (non-closure group).

There was no adverse outcome noted as a result of the study intervention. The operative times from skin incision to skin closure were not different between the groups (closure group median 25 min (range 12–38) vs. non-closure group median 23 min (range 14–51), p = .35).

## Discussion

This study indicates that suture closure of the subcutaneous fat at the time of CS does not influence objective or subjective cosmetic appearance of the CS scar at six months postpartum.

Four previous studies have addressed the effect of suture closure of the subcutaneous fat on wound cosmesis after CS [9]–[12]. The only two studies available at the time of conception of our study had several methodological limitations including the use of a non-validated or undetermined scar evaluation tool, absent or undetermined observer blinding and scar assessment only six weeks or four months postoperatively [10], [12]. Two other studies were published after our study was initiated [9], [11]. Huppelschoten et al. [9] detected no difference, whereas de Graaf et al. [11] found that suture closure of the subcutaneous fat compared to non-closure was associated with worse cosmetic outcome. However, this result was based on a non-validated scar classification (ie, satisfactory versus not satisfactory).

In all but one [12] of these studies only elective CS were enrolled. We included all indications for cesarean section, including emergency cesarean section, making our results more generalizable. It has been emphasized that the use of validated scar assessment tools is of great importance to produce comparable and valid results [25], [26]. Therefore we used the POSAS, an internationally developed and validated assessment tool for linear scar evaluation, as our primary outcome measure. It includes patient self-assessment of scar-related symptoms and physical characteristics and is considered the most suitable of the available linear scar rating tools [21], [27]. We chose six months as follow-up time for the final scar analysis according to previous studies investigating the long-term cosmetic outcome of linear surgical scars. Significant improvement of scar cosmesis occurs up to six months postoperatively, whereas no further improvement occurs after 12 months [28]–[30]. The remodeling processes that occur up to six months are documented by our finding that POSAS summary scores improved significantly between the two and six months follow-up.

We found that suture closure of the subcutaneous fat resulted in significantly less wound hematomas. Hematomas predispose to wound morbidity, often represent major concerns to the patient and are therefore to be avoided. Our findings correspond with previous studies investigating the effect of suture closure of the subcutaneous tissue in obstetrical patients, although the rate of wound hematomas in our trial was slightly higher [7]. Objective evaluation of hematomas is difficult due to the lack of a validated evaluation or measurement tool. We defined hematoma as any visible or otherwise diagnosed (ie, ultrasound) collection of blood surrounding the wound to minimize subjectivity. This strict definition most likely explains the increased hematoma rate in our trial. We found no difference regarding the rate of SSI or wound disruption between both groups. However, our study was not powered to identify differences in these relatively rare outcome measures.

We acknowledge a number of limitations of our study. First, although the rate of women who were lost to follow-up (21,6%) was less than expected and comparable to similar studies in the literature [6], [9], [10] it was still significant. However, there was no difference regarding any demographic or obstetric characteristics or intra- or postoperative complications in patients lost to follow-up compared to patients who completed the trial. Second, we did not measure the thickness of the subcutaneous tissue at the time of surgery. We acknowledge that many clinicians routinely close the subcutaneous tissue when it is larger than two centimeters to reduce the risk of wound disruption and recognize that this may be a potential confounder [8]. However the primary outcome measure of this trial was cosmetic outcome and not wound disruption. Wound disruption may lead to poor cosmetic results, but equally closure of subcutaneous tissue in itself leads to an inflammatory reaction against the suture material and could therefore result in poorer cosmesis. In clinical reality the subcutaneous tissue will not always be measured with a ruler. Therefore, in some instances women with less than two centimeters subcutaneous fat might also receive suture closure. To assess the impact of subcutaneous suture closure on wound cosmesis in women with thick and thin subcutaneous layer, we randomized participating women without knowledge of the actual thickness in centimeters. The BMI scores were not significantly different between our two study groups and therefore the likelihood that the subcutaneous thickness (ie, more or less than two centimeters) was different between the groups is low. Since there was only one case of wound disruption in this trial the risk of a potential confounding effect of this complication on the interpretation of our results is also low. Third, we only included women without previous lower abdominal incisions. Women with previous CS represent a population that still needs to be studied. Last, the study was performed at a large teaching hospital with several surgeons performing the procedure, which may have influenced internal validity. However, because the intervention was standardized and simple, we think that the risk was generally small. On the other hand numerous surgeons performing the same procedure at one institution is common, which increases external validity of our study.

In conclusion, the results of this randomized trial suggest that suture closure of the subcutaneous fat compared to non-closure at the time of CS does not influence long-term cosmetic outcome. However, since suture closure reduces wound hematomas, has a neutral effect on cosmesis and previously was demonstrated to reduce wound disruption in women with subcutaneous fat larger than two centimeters [8], this trial supports a low threshold for suture closure of the subcutaneous fat at the time of CS. Further the results reported in this paper support estimation of the subcutaneous thickness as opposed to actual measurement, since subcutaneous suture closure does not negatively effect wound cosmesis.

## Supporting Information

S1 Checklist **CONSORT Checklist.**
(DOC)

S1 Data **Spreadsheet with the data underlying the results reported in the manuscript.**
(XLS)

S1 Protocol **Trial protocol.**
(DOCX)
