# Supplementary material for: Suture Closure versus Non-Closure of Subcutaneous Fat and Cosmetic Outcome after Cesarean Section: A Randomized Controlled Trial
Source: PLoS One. 2014 Dec 10;9(12):e114730. doi: 10.1371/journal.pone.0114730 (PMC4262443; doi:10.1371/journal.pone.0114730)
Supplement: S1 Protocol — Trial protocol. (DOCX) [file pone.0114730.s003.docx]

Study protocol:

Prospective randomized trial to assess the effect of suture closure of the subcutaneous tissue on cosmetic outcome after cesarean section

H. Husslein, B. Mathis, C. Herbst C. Worda, H. Leipold, S. Szalay

Dr. Heinrich Husslein

Klinikum-Klagenfurt am Wörthersee

Gynäkologisch-Geburtshilfliche Abteilung

St. Veiter Strasse 47

9020 Klagenfurt

Tel.: +43 463 538 26435

Introduction:

Within the last 15 years the cesarean section (CS) rate has increased dramaticaly. To this day CS is one of the most commonly performed major abdominal surgeries. In Austria the CS rate is currently varying between hospitals reaching from 25-50%.^[[1]](#endnote--1)^ Due to the large number CS performed each year, this operation gains importance.

Postoperative wound infection (or surgical site infection, SSI)) is one of the major contributors to postoperative morbidity after CS. Due to the increasing number of CS performed each year, preventing SSI is not only important for the patient but also because it significantly increases costs.

SSI after CS has been reported to range between 2,5% and 16%.^[[2]](#endnote-0)^ Risk factors include length of surgery, maternal BMI, diabetes mellitus, maternal age, and undernutrition.^[[3]](#endnote-1)^

Closure of the subcutaneous fat after CS is advocated by many authors to reduce SSI. However, there are also reports of increased wound infection after suture closure of the subcutaneous fat.^[[4]](#endnote-2)^ A recent Meta-analysis shows a 34% relative risk reduction of wound dehiscence in women whos subcutaneous fat is larger than 2 cm.^[[5]](#endnote-3)^ Although there seems to be a benefit in this subgroup of women, we observed a large variation in current routine application of suture closure of the subcutaneous fat in Austria and also internationally. In several discussions with physicians one argument that was repeatedly mentioned was that it is currently unknown if suture closure of the subcutaneous fat has an effect on cosmetic outcome. Many of these physicians believe it could worsen wound cosmesis and therefore don´t routinely close the subcutaneous tissue.

On the one hand, subcutaneous suture closure theoretically allows to perfectly allign the edges of the wound that needs to be closed. This would decrease tension on the skin an thereby promote better wound cosmesis. By closing off dead space it might also be possible to avoid retractions of the scar below the level of the skin; such retractions are espacially bothersome and apparent in the standing position. On the other hand, the suture material used for closure of the subcutaneous fat could lead to a foreign body reaction with increased inflammation and consequently negative effect on wound cosmesis

Study aim:

The aim of this study is to assess whether, single layer, interrupted closure of the subcutaneous fat rsults in better or worse subjective and objective cosmetic outcome after CS. Futher we want to assess the effects of suture closure of the subcutaneous fat on SSI development, seroma formation, wound hematoma formation and length of surgery.

Study design:

Prospective, randomized, double blind, contolled trial.

Study site:

Klinikum-Klagenfurt am Wörthersee

Gynäkologisch-Geburtshilfliche Abteilung

St. Veiter Strasse 47

9020 Klagenfurt

Outcome measures:

Primary Outcome:

Subjective and objective cosmetic outcome of the Pfannenstiel laparotomy. The methods used to assess cosmetic outcome are described in the section „protocol“. Further patient rated presence or absence of a scar retraction will be evaluated.

Secondary Outcome:

SSI, seroma formation, hematome formation and surgical length.

Study population:

Patients will be recruited in the prenatal clinic at the department of obstetrics and gynecology of the Klinikum Klagenfurt am Woerthersee.

Inclusion criteria:

- Minimum 18 years of age
- Younger than 50 years
- Study consent
- Literate in the german language

Exclusion criteria:

- Previous CS
- HELLP syndrome or preeclampsia
- Febrile infection 2 weeks prior
- Current signs of infection
- Chronic diseases which involve corticosteroid use
- Immunosupression
- History of keloids
- Allergy or sensitivity to used suture material

Number of patients:

To this point only one study that reported POSAS scores for CS scars was published. This study ^[[6]](#endnote-4)^ reported mean POSAS summary scores of 23.6 with a mean standard deviation of 7.6. Assuming a clinically significant effect would be evident if there is a 20% difference in mean POSAS summary scores and accounting for a mean standard deviation of 8, the sample size was calculated to be 41 participants for each arm applying α = 0.05 and 80% power. To account for an attrition rate of up to 40% a total of 116 patients were enrolled.

Randomization:

Patients will be randomized at the time of CS Indication. At the nursing station on the labor and delivery floor, a binder with opaque envelopes will be deposited. The envelopes contain the group allocation. In case of CS, the attending midwife draws the subsequent envelope, while the patient is being prepped for the surgery. Group allocation is then shown to the operator and OR nurse and not read out loud to maintain patient blinding.

Study-ID allocation:

Midwifes who have randomized a patient will bring a patient sticker to Mrs. Safron the administrative assistant of the chief of the department and she will allocate each participant an ID-number. The list (master linking log) will only be available to Mrs. Safron. For the follow-ups Mrs. Safron will be phoned to inquire the study-ID of a participant, which will allow correct data collection and allocation.

Abortion of study:

Participants can withdraw their consent to participate in this clinical trial at any time point without giving reasons. Withdrawal will not alter medical care or have any other negative effects.

Protocol:

1. Recruitment

Patients planning a delivery at our institution are typically seen around 37 weeks of gestation in prenatal clinics at our department. In this so called „first contact“ visit a thorough patient history is taken (including: parity, age, BMI prior and during the pregnancy, basic gynecologic history, previous surgeries, current and past medical history and current medications), HIV and Hep B status is evaluated, blood pressure and pulse are determined and a urine dip and sonography is performed. During sonography placental location and biophysical profile are assessed.

Patients will be asked to participate in this trial according to inclusion and exclusion criteria. If the patients is willing to participate after the trial was explained in full detail, she will sign the written inormed consent form. The patients chart will be marked with a colored paper, which will be available in the clinic rooms, so that she will be easily identifiable as a study participant. This way the attending personal at the time of delivery will recognize the patient to be a potential (in case of CS) study participant.

1. Operation

Patients who participate in the study who undergo a CS prior or after the onset of labor will be operated the following way, according to randomization:

**Group A**: The CS is performed according to the standards of our department. At the time of abdominal wall closure, prior to skin closure using staples, the subcutaneous tissue will be suture closed using 3-5 single interrupted Polysorb 3-0 sutures and a V26 needle. Goal is optimal leveling of the opposing wound sides, without putting to much tension on the knot to avoid necrosis.

**Group B:** The CS is performed according to the standards of our department. At the time of abdominal wall closure the subcutaneous tissue will not be closed prior to skin closure using staples.

At this point in time we do not measure the sucutaneous fat layer using a ruler at our department. Unfortunately our standard operating set for CS does not contain a ruler. Therefore we will not be able to measure the subcutaneous fat using a ruler during CS in this study.

The rest of the procedure and postoperative care are not affected due to study participation. Operative times (skin incision and finished wound closure) as well as the names of the operators are collected routinely. In the OR report the closure or non-closure of the subcutaneous fat will not be mentioned, to assure observer blinding at the follow-up visits. Currently, as a review of OR-reports of the last months showed, type of subcutaneous closure is not routinely documented at our department. This will be preserved for the duration of the study. After the study the type of subcutaneous closure should however be documented.

3.) Postoperative follow-up

Patients are blinded regarding their group allocation. Group allocation is not noted in the patient chart, but only on the master linking log. Therefore only the surgeons will be aware of the patients group allocation and they will be educated to keep group allocation secret unless clinically otherwise indicated. Other staff involved in patient care will not be able to ascertain patient group allocation.

Following the surgery daily wound controls are performed until discharge. Wound controls are performed by the attending staff physician during morning rounding in the usual manner. Signs of infection , presence of hematoma, wound secretion, and wound dehiscence are noted. Further temperature and vitals are checked on a regular basis. Signs of infection in or outside the operative field are worked up and documented in a standard fashion and do not deviate due to study participation (eg, urine dip stick, urine culture, C/P, ultrasound or computed tomography). On postoperative day 3 a complete blood count including C-reactive protein is routinely drawn.

Postoperative infections are defined according to the CDC guidelines.^3^ Wound dehiscence are defined as any skin separation that is large enough to admit a cotton-tipped swab. ^[[7]](#endnote-5)^Unfortunately we were not able to find a clear method for the evaluation or measurement of hematomas published in the literature. Therefore we define a hematoma as any visible or otherwise diagnosed collection of blood surrounding the wound.

Patients are discharged at postoperative day 5 according to the standards of our department. At this time patients will be given dates for their two follow-up appointments, as well as a number which they can call to change dates if necessary. In case a participant misses a Follow-up they will be contacted via phone to arrange a new date. If patients drop out, specific reasons should be inquired.

According to the CDC guidelines there is still no optimal method for the detection of SSI´s after hospital discharge. However according to a study by Whtiby et al. ^[[8]](#endnote-6)^ patient reported SSI has a low positive predictive value, but a negative predictive value of 98%. Therefore it is assumed that almost all SSi´s will be detected even after discharge. An overview regarding SSI definitions will be given to the patient at the time of discharge.

Each participant will be seen 2 and 6 months after surgery. At the 2 month visit, initial wound cosmesis will be evaluated and data on SSI or other wound complications, including wound dehiscence will be collected. Final cosmetic outcome will be evaluated at the 6 month visit.

At both postoperative visits the CS scar will be evaluated using the following tools and scales:

1. Objective scar assessment using the Vancouver scar scale (VSS) ^[[9]](#endnote-7)^ ^[[10]](#endnote-8)^ and the observer component of the Patient and Observer Scar Assessment Scale (POSAS) ^[[11]](#endnote-9)^ ^[[12]](#endnote-10)^
2. Subjective scar assessment using the patient component of the POSAS
3. There is no validated method to assess scar retractions below the level of the skin. Therefore we will ask patients whether they feel that there scar is retracted below the level of the skin or not.
4. In case scar retractions are so pronounced that they can be measured with a ruler this will be done in order to quantify them.

Scar assessment will be performed by one of two available obervers. The observers are blinded to the patients group allocation, as is the patient. To be able to maintain blinding at all times, two available observers are necessary in case one of the observers was involved in the CS of the patient. These two observers will never perform a CS together due to internal logistics of the department.

Data collection:

All data will be entered direcly into a n excel spread sheet. This spread sheet will be stored on the F-drive and is available on each of the computers at the department. Data will be entered under the study-ID of the patient and the appropriate study visit (postop day 1-5 and follow-up visit after 2 and 6 months). Data regarding indication for CS and data regarding the surgery will also be entered this way. A backup of this excel spread sheet will be made each day. The scales which will be used to asses the scar will be available in form of case report forms (CRF) in the room which will be used for the follow-up visits (initial contact room on the labor and delivery floor). This data will be entered into the excel spread sheet secondary. The CRF will be given to Mrs. Safron who will archive it. On each CRF the participants study ID is noted.

Statistical analysis:

Statistical analysis will be performed according to the per protocol principle with SPSS software (version 21.0; SPSS, Chicago, IL). Continuous variables will be summarized as median (range), and categorical data as percentages. Skewed distribution will be tested via Kolmogrov Smirnov test. Chi2 test and Mann-Whitney-U test will be used accordingly. P values of ≤.05 will be considered to be significant.

References:

1. Geburtenregister – IET Institut für klin. Epidemiologie der Tilak [↑](#endnote-ref--1)
2. Owen J,Andrews W.Woundcomplicationsaftercesar- ean section. Clin Obstet Gynecol 1994;37:842–55. [↑](#endnote-ref-0)
3. Mangram AJ, Horan TC, Pearson ML, Silver LC, Jarvis WR. Guidelines for prevention of surgical site infection, 1999. Infect Control Hosp Epidemiol 1999;20:250–78. [↑](#endnote-ref-1)
4. De Holl D, Redeheaver G, Edgerton MT, Edlich RF. Potentiation of infection by suture closure of dead space. Am J Surg 1974;127:716–20. [↑](#endnote-ref-2)
5. David Chelmow, MD, Elisa J. Rodriguez, MD, and Marie M. Sabatini, MD. Suture Closure of Subcutaneous Fat and Wound Disruption After Cesarean Delivery: A Meta-Analysis. Obstet Gynecol 2004;103:974 – 80. [↑](#endnote-ref-3)
6. Cromi A, Ghezzi F, Gottardi A, Cherubino M, Uccella S, Valdatta L. Cosmetic outcomes of various skin closure methods following cesarean delivery: a randomized trial. Am J Obstet Gynecol 2010;203:36.e1-8. [↑](#endnote-ref-4)
7. Chelmow D, Huang E, Strohbehn K. Closure of the subcutaneous dead space and wound disruption after Cesarean delivery. J Matern Fetal Neonatal Med 2002;11(6):403-8. [↑](#endnote-ref-5)
8. Whitby M. et al. Post- Discharge Surveillance: Can Patiens Reliably Diagnose Surgical Wound Infetion? J Hosp Infect 2002; 52: 155 – 160 [↑](#endnote-ref-6)
9. Sullivan T, Smith J, Kermode J, McIver E, Courtemanche DJ. Rating the burn scar. J Burn Care Rehabil 1990;11:256-60. [↑](#endnote-ref-7)
10. Baryza MJ, Baryza GA. The Vancouver Scar Scale: an administration tool and its interrater re- liability. J Burn Care Rehabil 1995;16:535-8. [↑](#endnote-ref-8)
11. Draaijers LJ, Tempelman FR, Botman YA, et al. The patient and observer scar assess- ment scale: a reliable and feasible tool for scar evaluation. Plast Reconstr Surg 2004;113: 1960-5. [↑](#endnote-ref-9)
12. van de Kar AL, Corion LU, Smeulders MJ, Draaijers LJ, van der Horst CM, van Zuijlen PP. Reliable and feasible evaluation of linear scars by the Patient and Observer Scar Assessment Scale. Plast Reconstr Surg 2005;116:514-22. [↑](#endnote-ref-10)
